# Cardiopulmonary Arrest with Airway Obstruction due to Postoperative Bleeding

**DOI:** 10.1155/2021/8861061

**Published:** 2021-01-15

**Authors:** Kenichi Sato, Mami Chikuda, Yoshihisa Miyamae, Miwako Kan

**Affiliations:** Division of Dental Anesthesiology, Department of Reconstructive Oral and Maxillofacial Surgery, School of Dentistry, Iwate Medical University, 19-1 Uchimaru, Morioka, Iwate 020-8505, Japan

## Abstract

An 84-year-old woman underwent soft palate resection and skin grafting with tie-over under general anesthesia. Fourteen years previously, she had undergone aortic valve replacement and coronary artery bypass grafting followed by lifelong warfarin and aspirin anticoagulation. We terminated the two drugs 8 and 6 days, respectively, before the present surgery and substituted intravenous heparin (10,000 units/day), which was terminated 6 h preoperatively. The surgery was uneventful. Heparin was restarted 2 days postoperatively but without warfarin potassium or aspirin because of postoperative soft palate bleeding, which continued for 10 days despite compression hemostasis. On day 10, she exhibited a suffocating large hemorrhagic mass, leading to cardiopulmonary arrest. Emergency consultation with medical doctors and dental anesthetists resulted in pulmonary resuscitation and tracheal intubation. After confirming spontaneous circulation/respiration, she was transferred to the intensive care unit. We now consider it essential that all medical/surgical/anesthesia specialists managing patients under anticoagulant therapy collaborate perioperatively.

## 1. Introduction

Today's aging society has produced a growing population of patients on antithrombotic drugs for cerebrovascular and/or cardiovascular disease who are undergoing dental procedures. Guidelines [1] for anticoagulant and antiplatelet therapy for cardiovascular diseases recommend that antithrombotic drugs be changed to heparin sodium for perioperative management. We have often had difficulty, however, with perioperative hemostasis when administering treatments that themselves cause bleeding in patients taking antithrombotic drugs [2, 3]. Discontinuing or reducing anticoagulants (e.g., warfarin potassium) to avoid bleeding complications increases coagulation ability, thereby increasing the risk of developing thrombosis and/or embolism [4].

Because reducing the dosage of antithrombotic drugs or discontinuing them entirely, even for a short time, increases the risk of thrombogenesis, we must shorten the interruption period as much as possible or maintain anticoagulant conditions by other means [5]. The Iwate Medical University guidelines [6] recommend that the antithrombotic drugs in use be changed to heparin sodium to allow perioperative management for patients in whom postoperative bleeding would normally be expected. Some patients in whom the antithrombotic drug was changed to heparin sodium during the perioperative period, however, had to continue on heparin because of unremitting postoperative bleeding, precluding the switch back to their usual antithrombotic drug. We report such a patient in whom the situation led to airway obstruction and cardiopulmonary arrest because of hemorrhaging from the surgical wound 10 days after the surgery.

## 2. Case Presentation

The patient was an 84-year-old woman (height 148 cm, weight 39 kg). Left-palate tumor resection was scheduled under general anesthesia. Her history included the following events.

- At age 60: percutaneous transluminal coronary angiography for angina
- At age 65: plastic surgery for internal carotid artery stenosis; resection of a tongue tumor
- At age 70: coronary artery bypass and aortic valve replacement for aortic valve stenosis; surgery for varicose veins in the lower limbs; cataract surgery
- At age 84 (currently): under surveillance for a thoracic aortic aneurysm and a goiter mass

She was currently taking the following oral medications: aspirin, warfarin potassium, spironolactone, isosorbide nitrate, nitroglycerin, etizolam, and eszopiclone.

Electrocardiography showed complete right bundle branch block and left atrial enlargement. Echocardiography revealed mitral valve reflux disease and tricuspid valve dysplasia. The ejection fraction was 71%. Other hematologic tests, respiratory function tests, and chest radiography showed no abnormalities. She exhibited no chest pain or symptoms of heart failure, and her heart function was judged to be good.

The oral surgeon consulted her primary doctor and received instructions to discontinue the aspirin 8 days and the warfarin 6 days before the planned surgery. Thus, at the time of discontinuing the warfarin, we initiated heparin sodium (10,000 units/day) using a 24 h continuous drip, which was discontinued 6 h before commencing the operation [6]. On the day of surgery, the prothrombin time-international normalized ratio (PT-INR) was 1.02, and the activated partial thromboplastin time (APTT) was 36.0 s.

Rapid induction of anesthesia was undertaken with fentanyl citrate, midazolam, and rocuronium, followed by oral intubation. The anesthesia was maintained with air, oxygen, and sevoflurane. The surgical procedure involved the left side of the hard palate, soft palate, and cheek mucosa. The front and rear portions were sewn down, the central portion was implanted with Terdermis® (Terumo, Tokyo, Japan), and terramycin ointment gauze was placed on the Terdermis and the distal end of the soft palate. The tie-over was then completed. The operation time was 115 min, anesthesia time was 245 min, and the bleeding volume was 26 g.

During the operation, the patient's blood pressure occasionally dropped, and we took measures to increase it, as appropriate. After the patient awakened and wound hemostasis was confirmed, the tracheal tube was removed, and she was returned to the ward. On the day after the operation, however, continuous bleeding was apparent, and she was treated with compression hemostasis because her blood pressure and hematologic tests were normal. On postoperative day (POD) 2, after confirming wound hemostasis, heparin sodium (10,000 units/day) administration was resumed. Although bleeding from the wound again became evident, warfarin potassium (1.75 mg) was resumed on POD 3 and aspirin (100 mg) on POD 4. Because the bleeding had not stopped by POD 5, however, the warfarin and aspirin were discontinued. Heparin sodium (10,000 units/day in a sustained drip) was resumed, but continuous bleeding was seen through PODs 6–10, even though the patient was still being treated with compression hemostasis. Her APTT and PT-INR had been assessed both preoperatively and perioperatively (Table 1).

On POD 10, the oral surgeon, who routinely performed disinfection procedures on the dental unit of the ward, found a large amount of clotted blood in our patient that was obstructing her airways and making it difficult to breathe. There was an immediate emergency call, and experts from the medical emergency and dental anesthesia departments responded. Respiratory arrest occurred 7 min after discovering the clotted blood, and cardiopulmonary arrest was confirmed 10 min afterward. Cardiopulmonary resuscitation was started. A large amount of clotted material was found in the oral cavity, making tracheal intubation difficult. It was finally performed using the McGrath® apparatus, and 3 ampoules of adrenaline were administered intravenously.

The first rhythm check after tracheal intubation confirmed self-initiated resumption of the heartbeats and 100% SpO_2_ with auxiliary breathing using an ambu bag. Her blood pressure was 181/99 mmHg. Electrocardiography showed sinus rhythm, complete right bundle branch block, and obvious ST changes. The right pupil was 2.5 mm and the left was 3.0 mm. Light reflection was left and right quickly. Consciousness, according to the Glasgow Coma Scale, was 3 (eye opening 1, verbal response 1, and motor response 1), and she was transferred to the intensive care unit. At this point, heparin sodium administration was discontinued. Communication became possible on POD 14.

Because bleeding from the wound continued, tracheotomy was performed, and hemostatic sutures were placed under general anesthesia on POD 15. She was discharged from the hospital in good condition 5 months after the original surgery.

## 3. Discussion and Conclusion

Dental surgeons treating patients who are on antithrombotic medications should ensure that the patient undergoes preoperative coagulation function tests under the direction of the primary physician. Informed decisions can then be made on the basis of the laboratory results, the dental treatment proposed, and a thorough evaluation of the methods available for hemostasis.

One study reported that about 1% of cases of severe cerebral infarction occurred due to only a 1 h interruption of warfarin potassium administration [7]. The incidence of cerebral infarction was 3.4 times higher than usual if an aspirin regimen to prevent recurrence was interrupted [8]. In general, it is necessary to switch to heparin sodium therapy when the risk of thrombosis is high in a patient for whom the operation is predicted to cause a large amount of hemorrhaging and warfarin potassium administration cannot be interrupted.

The Iwate Medical University guidelines [6] for anticoagulant and antiplatelet therapy in patients with a cardiovascular disease who are undergoing major surgery are as follows. Warfarin potassium should be discontinued 3–5 days prior to surgery and changed to heparin sodium, which has a short half-life. The dosage should be adjusted, so the partial thromboplastin time extends to 1.5–2.5 times the normal control value. The heparin sodium is stopped 4–6 h before surgery or heparin sodium with protamine sulfate just before surgery. After the operation, warfarin potassium is resumed whenever possible, and heparin sodium administration continues until the PT-INR value is restored to within the proper range. Oral maxillofacial surgeons and other medical doctors refer to this Iwate Medical University guideline [6], which was designed with reference to the guidelines [1] established for anticoagulant and antiplatelet therapy for cardiovascular diseases.

Recent publications have indicated that perioperative heparin bridging anticoagulation increased the bleeding risk without decreasing the thromboembolic risk in patients. It has been reported that low-dose heparin bridging anticoagulation is unlikely to affect either bleeding or thromboembolic risks in patients undergoing major abdominal surgery for a malignancy [9]. It has also been reported that bridging with heparin in patients with atrial fibrillation is associated with a significant bleeding risk compared with not bridging [10]. Thus, bridging therapy seems controversial as it may not lower the risk of bleeding or thromboembolism.

Table 1 shows the APTT and PT-INR values on POD 1, the day of the operation, and PODs 2, 3, 5, 8, and 10. Thus, although our patient's APTT and PT-INR were measured every morning from the time of her hospitalization 7 days prior to the incident until POD 10, we failed to assess and cope with adequately what the results had suggested. Proper monitoring and assessment might have improved the outcome.

In the present case, when the warfarin potassium was switched to heparin sodium during the perioperative period, blood clotting occurred due to airway obstruction, leading to cardiopulmonary arrest due to postoperative hemorrhage. It was thought that aggressive hemostatic treatment was necessary at an early stage when continuous bleeding was confirmed from the start of heparin sodium administration. The cause of the postoperative bleeding was eliminated from the soft palate to the cheek mucosa by implementing a tie-over technique, while maintaining the range of motion. Although the soft palate and part of the cheek mucosa had not completely healed, there was sufficient hemostasis. Despite continuous bleeding, however, it was treated only with compression hemostasis, so blood clots were more likely to flow into the pharynx, blocking the airway and causing breathing difficulty. Fortunately, routine morning disinfection treatment by an oral surgeon identified a blood clot causing airway obstruction and difficult breathing. An emergency call from the oral surgeon gathered emergency doctors and dental anesthesiologists for a rapid response.

Oral surgeons must always keep in mind that a postoperative bleeding surgical site in the upper respiratory tract can lead to serious airway obstruction or breathing difficulty [2, 11], which requires appropriate management. To prevent such complications as postoperative bleeding, it is important for all those involved with a patient undergoing upper respiratory tract surgery to work together to plan careful perioperative management. As oral surgery for patients undergoing antithrombotic therapy continues to increase, oral surgeons must work closely with their patients' primary physicians and dental anesthesiologists to perform appropriate perioperative management, thereby preventing complications associated with postoperative bleeding.

We experienced a case of airway obstruction and cardiopulmonary arrest due to continuous postoperative bleeding after resecting a soft palate tumor. During the perioperative period, the patient's antithrombotic drugs had been replaced by sodium heparin. For patients at risk of bleeding complications, oral surgeons, physicians, and dental anesthesiologists should work together to ensure adequate perioperative management.

## Figures and Tables

**Table 1:** APTT and PT-INR values the day before, the day of, and several days after the operation.

	Day before operation	Day of operation	Days after operation				
	1	Before op.	2	3	5	8	10
APTT	100.9	36.0	85.6	68.0	72.3	53.0	57.1
PT-INR	1.05	1.02	0.98	0.93	0.97	0.91	0.98

The results for APTT measurements are expressed in seconds. APTT: activated partial thromboplastin time; PT-INR: prothrombin time-international normalized ratio.

## Additional Points

*Summary*. (i) An 84-year-old woman with a history of cardiac problems—and thus on a lifelong warfarin and aspirin regimen—required dental surgery that entailed palatal resection and skin grafting. (ii) Soft palate bleeding continued up to 10 days postoperatively because her anticoagulant regimen was problematic. (iii) On POD 10, an oral surgeon—routinely performing disinfection procedures on the dental unit of the ward—found that the patient had accumulated blood clots that were suffocating her, which led to cardiopulmonary arrest.
